# Decreased CSF Levels of ß-Amyloid in Patients With Cortical Superficial Siderosis

**DOI:** 10.3389/fneur.2019.00439

**Published:** 2019-04-26

**Authors:** Cihan Catak, Marialuisa Zedde, Rainer Malik, Daniel Janowitz, Vivian Soric, Anna Seegerer, Alexander Krebs, Marco Düring, Christian Opherk, Jennifer Linn, Frank A. Wollenweber

**Affiliations:** ^1^Institute for Stroke and Dementia Research, University Hospital, LMU Munich, Munich, Germany; ^2^Neurology Unit, Stroke Unit, Arcispedale Santa Maria Nuova, Azienda Unità Sanitaria Locale-IRCCS Reggio Emilia, Reggio Emilia, Italy; ^3^MVZ Labor PD Dr. Volkmann und Kollegen, Gesellschaft Bürgerlichen Rechts, Karlsruhe, Germany; ^4^Klinik für Neurologie, SLK-Kliniken Heilbronn GmbH, Heilbronn, Germany; ^5^Institut und Poliklinik für Neuroradiologie, University Hospital Carl Gustav Carus, Dresden, Germany

**Keywords:** cerebral amyloid angiopathy, cortical superficial siderosis, cerebrospinal fluid, cerebral microbleeds, neuroimaging

## Abstract

**Background:** Cortical superficial siderosis (cSS) represents a key neuroimaging marker of cerebral amyloid angiopathy (CAA) that is associated with intracranial hemorrhages and cognitive impairment. Nevertheless, the association between cSS and core cerebrospinal fluid (CSF) biomarkers for dementia remain unclear.

**Methods:** One hundred and one patients with probable (79%, 80/101) or possible (21%, 21/101) CAA according to the modified Boston criteria and mild cognitive impairment according to Petersen criteria were prospectively included between 2011 and 2016. CSF analyses of ß-amyloid 42, ß-amyloid 40, total tau and phosphorylated tau were performed using sandwich-type enzyme-linked immunosorbent-assay. All patients received MRI and Mini-Mental-State Examination (MMSE). Logistic regression analysis was used to adjust for possible confounders.

**Results:** cSS was present in 61% (62/101). Of those, 53% (33/62) had disseminated cSS and 47% (29/62) focal cSS. ß-amyloid 42 was lower in patients with cSS than in patients without cSS (OR 0.2; 95% CI 0.08–0.6; *p* = 0.0052) and lower in patients with disseminated cSS than in those with focal cSS (OR 0.02; 95% CI 0.003–0.2; *p* = 0.00057). Presence of cSS had no association with regard to ß-amyloid 40, total tau and phosphorylated tau.

**Conclusions:** Our results demonstrate that the presence and extent of cSS are associated with reduced CSF ß-amyloid 42 levels. Further studies are needed to investigate the underlying mechanisms of this association.

## Introduction

Cerebral amyloid angiopathy (CAA)—characterized by the deposition of ß-amyloid in the walls of leptomeningeal vessels—is a common cerebral small vessel disease and a major cause of intracerebral hemorrhage in the elderly (1–3). Furthermore, it has become evident that CAA is associated with cognitive impairment (4). Specifically, it has been shown that CAA patients perform worse in executive functioning, perceptual speed, and episodic memory compared with normative values (5).

The various CAA-related MRI structural lesions essentially represent vascular-mediated brain damage, rather than abnormalities of CAA-laden vessels themselves. CSF biomarkers that are typically altered in AD [ß-amyloid 42 (Aß42), ß-amyloid 40 (Aß40), total tau (t-tau) and phosphorylated tau (p-tau)] may offer another approach to understand underlying mechanisms and courses of patients with CAA (6). While Aß42 can be detected in both senile plaques of AD patients and capillaries of CAA patients, Aß40 deposition is supposed to appear preferentially in the walls of leptomeningeal arteries of CAA patients (7, 8).

More recently, it has been suggested that CAA-related cognitive impairment is more pronounced in a subgroup of CAA patients that demonstrate the MRI marker “cortical superficial siderosis” (cSS), which most likely reflect blood residues in the subarachnoid space (9). In line with this, patients from memory clinic populations have a higher cSS prevalence of 2–6% (10–12) compared to the general population at around 1% (13).

We hypothesized that differences between cSS positive and cSS negative patients might also be reflected in different CSF biomarker profiles. Since cSS patients are regarded to be of a higher risk for future neurovascular events and for cognitive dysfunction, we hypothesized that cSS positive patients would demonstrate lower levels of Aß40 and Aß42 together with elevated tau levels. Hence, we analyzed the core CSF biomarker profile in a prospective cohort of patients with mild cognitive impairment and the diagnosis of a possible or probable CAA.

## Methods

### Subjects

Screening took place in the outpatient clinics of the university hospitals in Reggio Emilia (Italy) and LMU Munich (Germany). Three hundred and forty-five patients with subjective cognitive impairment and the MRI based suspicion of a CAA were screened. Of those 71% (244/345) did not undergo lumbar puncture because of (a) normal objective cognitive testing (*n* = 134), (b) patient decline (*n* = 90) or (c) intake of oral anticoagulation (*n* = 20). The remaining 101 patients entered the final analysis. Seventy-nine percent (80/101) had a probable CAA and 21% (21/101) a possible CAA according to the modified Boston criteria (2). All patients in the final analysis had the diagnosis of mild cognitive impairment based on the Petersen criteria (14). The reasons for the initial presentation at the outpatient clinics were distributed as follows: past intracranial hemorrhage 34% (34/101), memory complaints 39% (39/101), past ischemic stroke or TIA 14% (14/101), transient focal neurological episodes (TFNE) or presumed focal seizure 4% (4/101), dizziness, headache or other unspecific neurological complaints 10% (10/101). Patients not included into the study did not differ in terms of age gender and cardiovascular risk factors (Supplementary Table 3). Informed consent were obtained from each patient according to the Declaration of Helsinki. Ethics approval was obtained from the regional ethics board.

### MRI

MR images in Munich were acquired on a 3 Tesla MRI scanner (Signa HDxt, GE Healthcare), in Reggio Emilia on a 1.5 Tesla scanner (Achieva, Philips Healthcare). Cortical superficial siderosis was identified on T2^*^-weighted gradient-echo sequence (GRE) or susceptibility-weighted imaging (SWI) by two trained raters (FAW and CC). Disagreement in two cases was resolved by consensus read (interrater κ = 0.97). Cerebral microbleeds (CMB) were defined according to the STRIVE criteria (15). CMB were manually marked on lesion masks, which were then normalized to 1 mm Montreal Neurological Institute (MNI) standard space and automatically rated as described before (16). CMB were categorized in 4 groups (0 CMB, 1 CMB, 2–4 CMB and ≥5 CMB) according to the recent literature (17, 18).

### CSF

CSF samples were obtained by lumbar puncture within 6 weeks after MRI, collected in 10 mL polypropylene tubes and centrifuged within 2 h, and then frozen until analysis. Aß42, Aß40, t-tau and p-tau were measured using sandwich type enzyme linked immunosorbent assays; Ab42 was measured with Innotest ß-amyloid (42), Ab40 was measured with Innotest ß-amyloid (40), t-tau with Innotest hTau-Ag, and p-tau with Innotest Phospho-tau (181 P) (Innogenetics, 9,052 Gent, Belgium, www.fujirebio-europe.com). The unit used for biomarkers is pg/mL.

The inter lot variation coefficient between the centers were Aß42 5.15%, Aß40 7.87%, t-tau 14.01%, p-tau 16.2% and Aß42 5.7%, Aß40 8.2%, t-tau 7.5, p-tau 16.4. Further, there were no significant differences between the median levels of core CFS markers between the two centers, indicating a good comparability of the CSF assays (Supplementary Table 4).

### Statistical Analysis

Baseline characteristics and risk factors were compared using chi-square tests, Fisher's exact tests and student's *t*-tests where appropriate. Logistic regression analyses corrected for age, sex and CMB category was performed using the cSS groups as dependent variables. To use the binary cSS status as the dependent variable in the logistic regression, the analysis was inverted. All CSF marker levels were log-transformed for regression analysis and are presented as median with interquartile range to account for the non-normal distribution. Logistic regression analyses with MMSE as the dependent variable were performed using logistic regression corrected for age, sex, hypertension, diabetes and hypercholesterinemia. MRI field strength and T2^*^-weighted method (GRE vs. SWI) were tested as a variable in univariate analysis. Therefore, it was not included into the logistic regression model. The relationship between cSS and the categories of CMB and CSF markers was tested using ordinal logistic regression under a proportional odds assumption. For the additional model, we applied a backward stepwise regression analysis optimizing on Akaike Information Criterion (AIC) to statistically select for relevant covariates. As a result, hypertension was selected as the only parameter. All analyses was performed using R (R version 3.5.1: A language and environment for statistical computing; R Foundation for Statistical Computing, Vienna, Austria).

## Results

CSS was present in 61% (62/101). Patients with and without cSS were well balanced with regard to age, sex, and cardiovascular risk factors. MRI field strength and T2^*^-weighted method (GRE vs SWI) did not show any significant effect on the presence and extent of cSS (all *p* > 0.4). Detailed baseline characteristics are shown in Tables 1, 2.

**Table 1:** Baseline characteristics stratified for the presence of cSS.

	**CAA (*n* = 101)**	**CAA with cSS (*n* = 62)**	**CAA without cSS (*n* = 39)**	***p***
Sex, male, *n* (%)	59 (58)	37 (60)	22 (56)	0.8
Age, mean, ± SD	76 ± 7	76 ± 7	75 ± 7	0.3
Hypertension, *n* (%)	69 (68)	40 (65)	29 (74)	0.4
Hypercholesterolemia, *n* (%)	41 (41)	23 (37)	18 (46)	0.4
Diabetes mellitus, *n* (%)	5 (5)	3 (5)	2 (5)	1
APOE ε2, *n* (%)	12 (12)	8 (13)	4 (10)	0.8
APOE ε4, *n* (%)	13 (13)	6 (10)	7 (18)	0.2
MMSE, median (IQR)	24 (18–28)	23 (17–26)	27 (21–29)	0.4
Number of CMB, *n* (%)				0.03
0	16 (16)	9 (15)	7 (18)	
1	12 (12)	8 (13)	4 (10)	
2–4	27 (27)	11 (18)	16 (41)	
≥5	39 (39)	30 (48)	9 (23)	

*cSS, cortical superficial siderosis; ApoE, Apolipoprotein E; CMB, cerebral microbleeds; SD, standard deviation*.

**Table 2:** Baseline characteristics stratified for extent of cSS.

	**Disseminated cSS (*n* = 33)**	**Focal cSS (*n* = 29)**	***P***
Sex, male, *n* (%)	18 (55)	19 (66)	0.4
Age, mean, y	76 ± 8	76 ± 5	0.3
Hypertension, *n* (%)	19 (58)	21 (72)	0.3
Hypercholesterolemia, *n* (%)	10 (30)	13 (45)	0.3
Diabetes mellitus, (%)	1 (3)	2 (7)	0.6
APOE ε2, *n* (%)	5 (15)	3 (10)	0.7
APOE ε4, *n* (%)	4 (12)	2 (7)	0.7
MMSE, median (IQR)	22 (15–26)	24 (19–27)	0.09
Number of CMB, *n* (%)			0.1
0	2 (6)	7 (24)	
1	3 (9)	5 (17)	
2–4	8 (24)	3 (10)	
≥5	17 (52)	13 (45)	

*cSS, cortical superficial siderosis; ApoE, Apolipoprotein E; CMB, cerebral microbleeds; SD, standard deviation*.

### Presence of cSS and MMSE

CAA patients with cSS had numerically lower median MMSE values than CAA patients without cSS (23 vs. 27, Table 1). However, this difference did not reach statistical significance, neither without adjustment (0.4), nor in a model adjusting for sex, age and cardiovascular risk factors (*p* = 0.05) nor in a model adjusting for hypertension only (*p* = 0.1).

### Presence of cSS and CSF Markers

**Figure 1:**
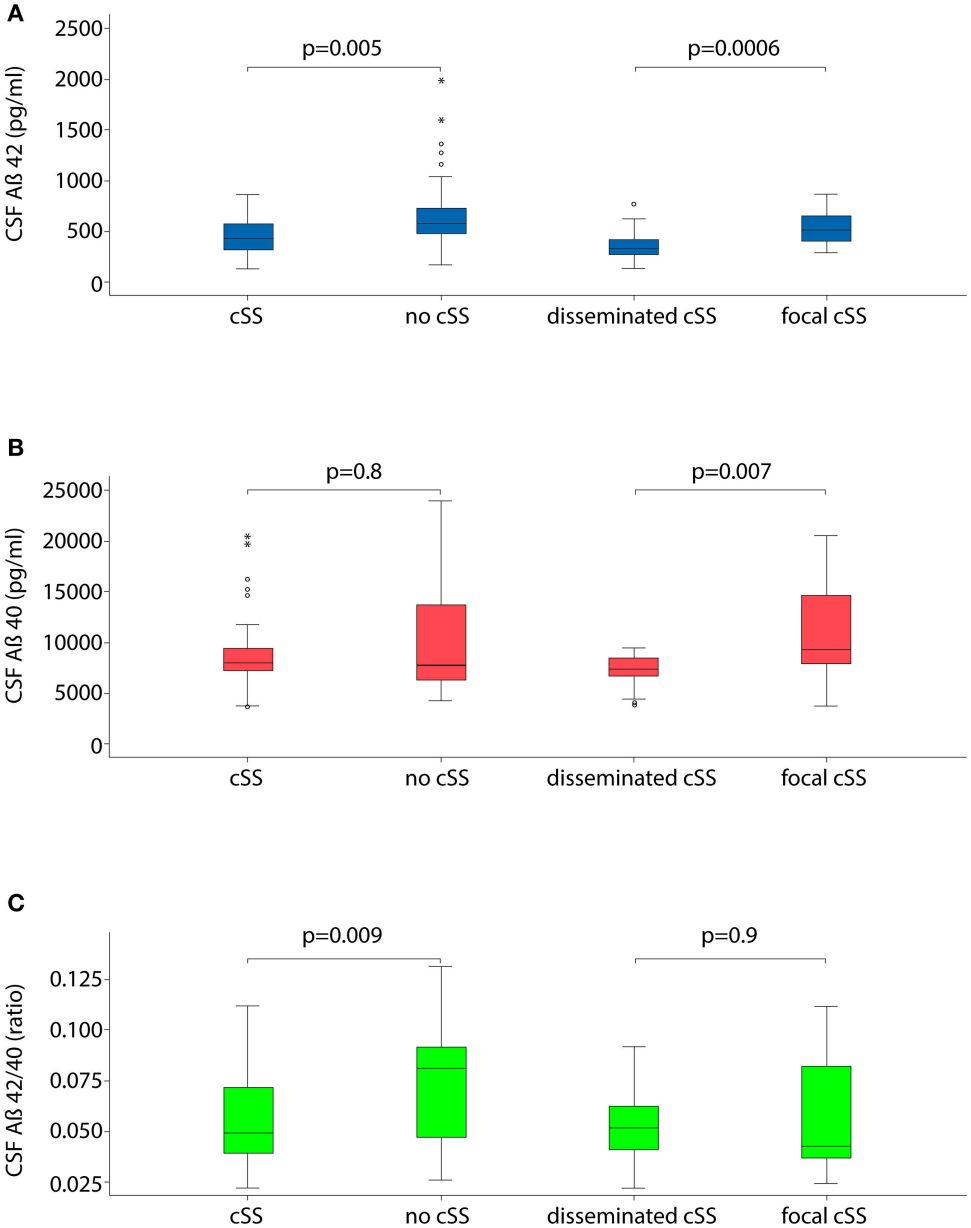
Boxplots illustrating cerebrospinal fluid (CSF) markers for dementia stratified for the presence and extent of cortical superficial siderosis (cSS). **(A)** CSF level of Aß42; **(B)** CSF level of Aß40; and **(C)** ratio of Aß42 to Aß40.

In logistic regression analyses, Aß42 was significantly lower in patients with cSS compared to those without (OR 0.2; 95% CI 0.08–0.7; *p* = 0.0052, Figure 1 and Table 3). Further, the Aß42/40 ratio was significant lower in patients with cSS than in patients without cSS (OR 0.3; 95% CI 0.08–0.7; *p* = 0.0091). Patients with and without cSS did not show significantly different CSF levels of Aß40 (OR 0.8; 95% CI 0.3–2.6; *p* = 0.77), t-tau (OR 1.9; 95% CI 0.8–4.5; *p* = 0.12), and p-tau (OR 2.1; 95% CI 0.8–5.3; *p* = 0.12).

**Table 3:** CSF marker stratified for presence of cSS.

	**Total (*n* = 101)**	**CSS (*n* = 62)**	**No cSS (*n* = 39)**	***P***	**OR (95% CI)**
Aß42 (pg/ml), median (IQR)	474 (352–629)	400 (326–566)	575 (474–730)	**0.005**	**0.2 [0.08–0.7]**
Aß40 (pg/ml), median IQR	8,079 (6,803–10,603) (*n* = 79)	8,170 (7,284–9,534) (*n* = 44)	7,906 (6,410–13,917) (*n* = 35)	0.8	0.8 [0.3–2.6]
T-tau (pg/ml), median (IQR)	322 (242–431)	319 (251–436)	331 (216–429)	0.1	1.9 [0.8–4.5]
P-tau (pg/ml), median (IQR)	58 (43–75)	60 (51–86)	50 (36–72)	0.1	2.1 [0.8–5.3]
Aß42/40 ratio, median (IQR)	0.06 (0.04–0.08) (*n* = 79)	0.05 (0.04–0.07) (*n* = 44)	0.08 (0.04–0.09) (*n* = 35)	**0.009**	**0.3 [0.08–0.7]**

*Logistic regression with adjustment for age, sex, number of cerebral microbleeds, MMSE, cholesterol, diabetes, and hypertension. All CSF marker levels were log-transformed for regression analysis. Odds Ratios correspond to one unit increase in log-transformed levels. Where data were missing; the number of subjects for which data were available is indicated within parentheses. SD, standard deviation; t-tau, total tau; p-tau, phosphorylated tau; Aß42, ß-amyloid 1–42; Aß40, ß-amyloid 1–40; cSS, cortical superficial siderosis; CSF, Cerebrospinal fluid; MMSE, Mini Mental State Examination; OR, odds ratio; CI, confidence interval. Significant values are indicated in bold*.

### Extent of cSS and CSF Markers

Out of the overall cohort, 33% (33/101) had disseminated (i. e. affecting >3 cortical sulci) cSS and 29% (29/101) had focal (affecting ≤ 3 sulci) cSS. CSF levels of Aß42 and Aß40 were significantly lower in patients with disseminated cSS than in focal cSS (OR 0.02; 95% CI 0.002–0.2; *p* = 0.00057 and OR 0.01; 95% CI 0.0003–0.3; *p* = 0.0069, please see Figure 1 and Table 4).

**Table 4:** CSF marker stratified for extent of cSS.

	**Disseminated cSS (*n* = 33)**	**Focal cSS (*n* = 29)**	***P*-value**	**OR (95% CI)**
Aß42 (pg/ml), median (IQR)	332 (269–421)	435 (367–679)	**0.0006**	**0.02 [0.002–0.2]**
Aß40 (pg/ml), median (IQR)	7,506 (6,784–8,901) (*n* = 23)	9,418 (7,986–14,621) (*n* = 21)	**0.007**	**0.01 [0.0003–0.3]**
T-tau (pg/ml), median (IQR)	322 (245–431)	309 (248–1214)	0.6	0.8 [0.3–2]
P-tau (pg/ml), median (IQR)	60 (51–67)	60 (51–90)	0.2	0.5 [0.1–1.6]
Aß42/40 ratio, median (IQR)	0.05 (0.04–0.06) (*n* = 23)	0.04 (0.04–0.08) (*n* = 21)	0.9	1.1 [0.2–4.9]

*Logistic regression with adjustment for age, sex, number of cerebral microbleeds, MMSE, cholesterol, diabetes and hypertension. All CSF marker levels were log-transformed for regression analysis. Odds Ratios correspond to one unit increase in log-transformed levels. Where data were missing, the number of subjects for which data were available is indicated within parentheses. SD, standard deviation; t-tau, total tau; p-tau, phosphorylated tau; Aß42, ß-amyloid 1–42; Aß40, ß-amyloid 1–40; cSS, cortical superficial siderosis; CSF, Cerebrospinal fluid; MMSE, Mini Mental State Examination; OR, odds ratio; CI, confidence interval. Significant values are indicated in bold*.

### CMB and CSF Marker

Eighty-four percent (85/101) of all patients had any CMB. Using Chi-Square test, there was a significant difference regarding the number of CMB in patients with and without cSS stratified for groups (*p* = 0.03). While patients with cSS less often had 2–4 CMB than patients without cSS (18%, 11/62 and 41%, 16/39), cSS patients were overrepresented in the group of >5 CMB (48%, 30/62 and 23%, 9/39). The number of CMB did not differ between patients with disseminated and focal cSS (*p* = 0.1).

In ordinal logistic regression analysis adjusting for age, sex and cardiovascular risk factors, there was no significant correlation between the number of CMB and all tested CSF parameters (all *p* > 0.05).

### cSS and CSF Markers in Subgroups

We repeated the logistic regression analyses restricted to patients with probable CAA according to the modified Boston criteria (Supplementary Tables 1, 2). In that analysis, Aß42 remained lower in patients with cSS compared to those without (OR 0.3; 95% CI 0.07–0.9; *p* = 0.05). Further, the Aß42/40 ratio was significantly lower in patients with cSS than in patients without cSS (OR 0.3; 95% CI 0.07–0.9; *p* = 0.04). Also patients with disseminated cSS still showed significantly lower levels of Aß42 (OR 0.02; 95% CI 0.002–0.3; *p* = 0.003) and lower levels of Aß40 (OR 0.0004; 95% CI 0.0000006–0.3; *p* = 0.01).

## Discussion

The main results from this cohort study of 101 patients with a possible or probable CAA and CSF samples are that (1) CSF Aß42 is significantly lower in patients with cSS than in patients without cSS, and (2) CSF Aß42 is significantly lower in patients with disseminated cSS (>3 sulci) than in CAA patients with focal cSS.

The current results indicate that both the presence and the extent of cSS are associated with lower Aß42 CSF levels. Since accumulating evidence suggests that the presence and extent of cSS might be a marker of CAA severity with higher recurrence rates of intracerebral hemorrhage (3, 16), it may be possible that Aß42 may also act as a biomarker of CAA severity. Previous data on Aß42 CSF levels in patients with CAA are relatively rare and limited in power. A recent meta-analysis on the topic identified five heterogeneous CAA cohorts that included overall 60 patients (6).

The underlying mechanism of the presumed association between cSS and Aß42 remain to be elucidated. However, our results are consistent with neuropathological studies that found Aß42 trapped in the cerebral vessels of CAA patients and therefore hampers the transport of Aß42 toward the cerebrospinal fluid. Also, *in vitro* data and PET studies suggest, that low Aβ42 in CSF of CAA patients is related to ß-amyloid deposition in the vessel walls and the adjacent brain parenchyma (19, 20). Further, experimental studies and neuropathological findings have demonstrated a high co-incidence between CAA and Alzheimer's disease (AD) (21, 22). Therefore, it may be speculated that the demonstrated reduced Aß42 levels may partly be explained by a higher rate of a co-incident AD in patients with cSS. However, we did not detect higher tau levels in cSS patients contradicting this hypothesis. Hence neuropathological studies are needed to disentangle the complex interplay between cSS and AD.

Patients with disseminated cSS had significantly lower Aß40 levels than those with focal cSS in our cohort. We are not aware of any other study that investigated the extent of cSS and its association with Aß40. However, this result is in line with a case series of 15 patients with hereditary cerebral hemorrhage with amyloidosis–Dutch type (HCHWA-D) that detected decreased CSF Aß40 levels in HCHWA-D patients in comparison to healthy controls (23). Further, it is in accordance with neuropathological reports demonstrating deposition of Aß40 within the vessel walls of CAA patients (19). Since patients with disseminated cSS most probably represent a group of more severely affected CAA patients, they may harbor more Aß40 depositions within the vessel wall and consecutively demonstrate lower CSF Aß40 levels.

The MMSE values of patients with cSS were numerically lower than in patients without cSS as hypothesized by previous findings that detected a higher prevalence of cSS patients in memory clinics (10, 11). However, this difference did not reach statistical significance. Hence, the results of the current study prevent meaningful conclusions on the extent and etiology of cognitive impairment in patients with cSS. Longitudinal studies with repeated extensive neuropsychological testing and cSS lesion-volume mapping are needed to disentangle the complex interplay of vascular pathology and ß-amyloid-pathology in cSS and CAA.

Of note, there was no consistent association between the presence of cSS and Aß40 in the current study, which may relate to the experimental finding that Aß42 deposits earlier in the vessel wall than Aß40 (23–25). Alternatively, it might be speculated from the odds ratio of 0.8 that slight Aß40 CSF levels differences are present, but the association did not reach statistical significance due to a lack of statistical power. We did not formally compare patients with focal cSS and no cSS due to the small sample sized within subgroups, but it also may be speculated that the effect of lower Aß40 values in cSS is primarily driven by the subgroup of patients with disseminated cSS rather than those with focal cSS. Further studies with larger sample size are needed to fully capture the effect of cSS subgroups and Aß40.

Interestingly, our results did not show an association between the number of CMB and reduced levels of Aß42 or Aß40 in contrast to other reports (23, 26, 27). This discrepancy may partly be related to differences in cohort characteristics since the prevalence of CMB was very high with 84% in this cohort resulting in a potential ceiling effect of the association.

Our study has several strengths including the prospective design, the standardized CSF sampling and analysis as well as the central MR imaging. Limitations include the use of two different MR scanners with different field strengths (3.0 or 1.5 Tesla), that may have had an impact on the number of CMB and potentially the extent of cSS. However, we included MRI field strength and the effect of T2^*^-weighted method (GRE vs. SWI) as a variable in univariate analysis were it did not show a significant association with CSF markers which makes a strong effect very unlikely. Further, there are no pathological proven CAA cases included. However, 79% of patients were diagnosed with probable CAA according to the modified Boston criteria, which have demonstrated an excellent sensitivity and specificity for CAA (2). Additionally, we repeated the logistic regression analysis on core CSF markers restricted to the subgroup of patients fulfilling the diagnosis of probable CAA with stable results. Finally, differences in medical history between patients might have confounded results in CSF analysis. However, in the backward stepwise regression analysis, only hypertension resulted relevant and a subgroup analysis stratified for differences in medical history was regarded not meaningful due to the limited sample sizes.

## Conclusions

The present study demonstrates for the first time that presence and extent of cSS are associated with lower Aß42 CSF levels. Our data suggest that Aß42 may act as an additional marker of CAA severity. Nevertheless, further studies are needed to proof these findings and to investigate its underlying mechanisms.

## Data Availability

All datasets generated for this study are included in the manuscript and/or the Supplementary Files.

## Consent for Publication

All authors approved the final manuscript and consent to its publication.

## Ethics Statement

Informed consent was obtained from each patient according to the Declaration of Helsinki. Ethics approval was obtained from the regional ethics board (Ethikkommission LMU Munich). Written informed consent was obtained from all patients.

## Author Contributions

CC and FW designed the study. CC, MZ, DJ, and VS acquired clinical, neuropsychological and CSF data. RM analyzed the data. AK, JL, CO, and MD interpreted the manuscript. CC and FW interpreted and drafted the manuscript. FW supervised and revised the manuscript. All authors read and approved the final manuscript.

### Conflict of Interest Statement

The authors declare that the research was conducted in the absence of any commercial or financial relationships that could be construed as a potential conflict of interest.

## Abbreviations

CAA: Cerebral amyloid angiopathy
cSS: Cortical superficial siderosis
CSF: Cerebrospinal fluid
Aß42: ß-amyloid 42
Aß40: ß-amyloid 40
t-tau: total tau
p-tau: phosphorylated tau
MMSE: Mini-Mental State Examination
AD: Alzheimer's disease
CMB: Cerebral microbleeds
SD: Standard deviation
OR: odds ratio
CI: confidence interval
y: years
DM: Diabetes mellitus
ApoE: Apolipoprotein E
IQR: interquartile range
HCHWA-D: hereditary cerebral hemorrhage with amyloidosis of the Dutch type.
