# Supplementary material for: Decreased CSF Levels of ß-Amyloid in Patients With Cortical Superficial Siderosis
Source: Front Neurol. 2019 Apr 26;10:439. doi: 10.3389/fneur.2019.00439 (PMC6498501; doi:10.3389/fneur.2019.00439)
Supplement: Supplementary file 1 [file Data_Sheet_1.docx]

**Table 1:** Subgroup probable CAA: CSF marker stratified for presence of cSS

|  | **total**  **(n= 80)** | **cSS**  **(n=53)** | **no cSS**  **(n=27)** | **p value** | **OR (95% CI)** |
| --- | --- | --- | --- | --- | --- |
| Aß42 (pg/ml), median (IQR) | 455 (324-609) | 400 (321-546) | 560 (397-675) | 0.05 | 0.3 [0.1-1] |
| Aß40 (pg/ml), median (IQR) | 7906 (6803-10128)  (n=63) | 8170 (7242-9497)  (n=38) | 6991 (6376-12040)  (n=25) | 0.8 | 1.2 [0.3-4.9] |
| t-tau (pg/ml), median (IQR) | 317 (242-438) | 319 (262-441) | 269 (211-421) | 0.05 | 1.01 [1-7.7] |
| p-tau (pg/ml), median (IQR) | 57 (41-76) | 60 (53-88) | 49 (36-65) | **0.02** | **3.5 [1.2-10.5]** |
| Aß42/40 ratio, median (IQR) | 0.05 (0.04-0.08)  (n=63) | 0.04 (0.04-0.07)  (n=38) | 0.07 (0.04-0.09)  (n=25) | **0.04** | **0.3 [0.07-0.9]** |

Logistic regression with adjustment for age, sex, number of cerebral microbleeds, MMSE, cholesterol, diabetes and hypertension. All CSF marker levels were log-transformed for regression analysis. Odds Ratios correspond to one unit increase in log-transformed levels.Where data were missing, the number of subjects for which data were available is indicated within parentheses

Abbreviations: SD standard deviation, t-tau total tau, p-tau phosphorylated tau, Aß42 ß-amyloid 1-42, Aß40 ß-amyloid 1-40, cSS cortical superficial siderosis, CSF Cerebrospinal fluid, MMSE Mini Mental State Examination, OR= odds ratio, CI = confidence interval

**Table 2:** Subgroup probable CAA: CSF marker stratified for extent of cSS

|  | **disseminated cSS**  **(n=31)** | **focal cSS**  **(n=22)** | **p value** | **OR (95% CI)** |
| --- | --- | --- | --- | --- |
| Aß42 (pg/ml), median (IQR) | 332 (274-438) | 435 (367-642) | **0.003** | **0.02 [0.002-0.3]** |
| Aß40 (pg/ml), median (IQR) | 7506 (6780-8904)  (n=21) | 9418 (7986-13199)  (n=17) | **0.01** | **0.0004 [<0.0001-0.3]** |
| t-tau (pg/ml), median (IQR) | 322 (257-435) | 309 (248-548) | 0.5 | 0.7 [0.3-1.9] |
| p-tau (pg/ml), median (IQR) | 60 (52-68) | 60 (53-100) | 0.2 | 0.4 [0.1-1.4] |
| Aß42/40 ratio, median (IQR) | 0.06 (0.04-0.07)  (n=21) | 0.04 (0.03-0.08)  (n=17) | 0.5 | 1.9 [0.3-10] |

Logistic regression with adjustment for age, sex, number of cerebral microbleeds, MMSE, cholesterol, diabetes and hypertension. All CSF marker levels were log-transformed for regression analysis. Odds Ratios correspond to one unit increase in log-transformed levels.Where data were missing, the number of subjects for which data were available is indicated within parentheses

Abbreviations: SD standard deviation, t-tau total tau, p-tau phosphorylated tau, Aß42 ß-amyloid 1-42, Aß40 ß-amyloid 1-40, cSS cortical superficial siderosis, CSF Cerebrospinal fluid, MMSE Mini Mental State Examination, OR= odds ratio, CI = confidence interval

**Table 3:** Baseline characteristics

|  | **not include total**  **(n=244)** | **include total**  **(n=101)** | **p value** |
| --- | --- | --- | --- |
| Sex, male, n (%) | 132 (54) | 59 (58) | 0.3 |
| Age, mean, + SD | 75 ± 7 | 76 ± 7 | 0.7 |
| Hypertension, n (%) | 149 (61) | 69 (68) | 0.1 |
| Hypercholesterolemia, n (%) | 85 (35) | 41 (41) | 0.2 |
| DM, n (%) | 12 (5) | 5 (5) | 0.9 |

Abbreviations: DM Diabetes mellitus, SD standard deviation

**Table 4:** Median and coefficient of variation of the two centers

|  | **center I** | **center II** | **p value** |
| --- | --- | --- | --- |
| Aß42 (pg/ml), median (cv) | 527 (5.2%) | 494 (5.7%) | 0.3 |
| Aß40 (pg/ml), median (cv) | 10934 (7.9%) | 10082 (8.2) | 0.1 |
| t-tau (pg/ml), median (cv) | 432 (14%) | 553 (7.5%) | 0.08 |
| p-tau (pg/ml), median (cv) | 55 (16.2%) | 58 (16.4%) | 0.2 |

Median and variation coefficient between the two centers, effects of site-to-site variations by univariate analyses Abbreviations: cv coefficient of variation, t-tau total tau, p-tau phosphorylated tau, Aß42 ß-amyloid 1-42, Aß40 ß-amyloid 1-40
